# Postoperative cognitive functions in patients with benign intracranial lesions

**DOI:** 10.1038/s41598-021-88061-6

**Published:** 2021-04-22

**Authors:** Stefanie Bette, Julia M. Ruhland, Benedikt Wiestler, Melanie Barz, Bernhard Meyer, Claus Zimmer, Yu-Mi Ryang, Florian Ringel, Jens Gempt

**Affiliations:** 1 Department of Neuroradiology, Klinikum Rechts Der Isar, Technische Universität München, Munich, Germany; 2 Department of Neurosurgery, Klinikum rechts der Isar, Technische Universität München, Ismaningerstr. 22, 81675 Munich, Germany; 3 Department of Neurosurgery, University Medical Centre, Johannes-Gutenberg-University Mainz, Mainz, Germany; 4 Department of Diagnostic and Interventional Radiology, Universitätsklinikum Augsburg, Augsburg, Germany

**Keywords:** Neurology, Oncology

## Abstract

The aim of this study was to assess pre- and postoperative cognitive functions in patients who underwent surgery for benign intracranial lesions. In total, 58 patients (21 men, 37 women, mean age 51.6 years [range 24–76 years]) with benign intracranial lesions (including benign tumors and vascular lesions) and neuralgia of the trigeminal nerve were included in this prospective study. Extensive cognitive testing was used to categorize *attention*, *memory*, and *executive functions*. Mood and pain were assessed preoperatively (t_0_, mean 3.7 days before surgery), immediately after surgery/during inpatient stay (t_1_, mean 7.6 days after surgery), and at first outpatient check-up (t_2_, mean 99.5 days after surgery). All 58 patients were tested at t_0_ and t_1_, but at t_2_ only 24 patients were available at t_2_. The data were categorized as improvement/stable condition or deterioration and shown as percentages. The pre- and postoperative values of BDI-II and mood were compared by the Wilcoxon test for paired samples. Binary logistic regression analyses were performed to identify parameters influencing cognition in the subgroup of meningioma patients. Immediately after surgery (t_1_), the percentage of patients with improvement/stable condition was > 50% in all categories in the majority of subtests (attention: 12/14 subtests, memory: 11/13 subtests, executive functions: 6/9 subtests). Similar results were shown at t_2_. Mood and pain did not change significantly after surgery. Factors like age, Karnofsky performance status, and tumor volume were not shown as significant influencing factors for cognitive functions in meningioma patients. The results of this study suggest that—in contrast to neuroepithelial tumors—cognitive functions do not deteriorate after surgery of benign intracranial lesions. Further studies are necessary to evaluate the results of this study.

## Introduction

Extensive cognitive testing is not used routinely before or after surgery of intracranial lesions. The main factors assessed in clinical routines and in neurooncological studies are age, functional independence (measured by the Karnofsky Performance Status Scale [KPS]^1^), neurological status, and extent of resection. However, evidence is increasing that cognition is important for not only quality of life but also overall survival^2–5^. Many studies have assessed pre- and postoperative cognitive functions in patients with gliomas and brain metastases^6–11^. Several studies have also evaluated the effect of meningioma surgery on cognitive functions^12–19^. Most of these studies have shown preoperative impairment and postoperative improvement of cognition in meningioma patients, sometimes with conflicting results^16–18,20^. Zweckberger et al.^19^ reported delayed improvement of cognitive functions, whereas another recent study showed ongoing impairment^21^. Little is known about pre- and postoperative cognitive functions in patients with rare intracranial tumors or vascular lesions like arterious venous malformations^22,23^. For unruptured aneurysms, the ISAT trial showed improved cognitive outcomes after endovascular treatment, as compared to a neurosurgical approach^24^.

Mood and pain are known to influence cognition^25,26^, so these measurements were also included in the study.

The aim of this study was to assess whether cognitive functions, mood, and pain improve or deteriorate after surgery in patients with benign intracranial lesions.

## Methods

### Study design

The local ethics committee approved this prospective single-center study (clinical trial registration number 3094/11). All participants signed an informed consent form. The study was conducted in accordance with the ethical standards of the 1964 Declaration of Helsinki and its later amendments^27^.

### Patient population

Patients who underwent surgery for a benign intracranial process (including benign tumors, vascular lesions, and neuralgia of the trigeminal nerve) between September 2012 and December 2014 were prospectively included in this study.

The data of the preoperative study population and the risk factors for preoperative cognitive impairment have been reported elsewhere^28^.

The following inclusion criteria were defined: age ≥ 18 years, informed consent, extensive neuropsychological testing of pre- and postoperative performance, surgery for a benign intracranial process, preoperative magnetic resonance imaging (MRI), knowledge of the German language, no pregnancy, and preoperative Mini-Mental Status Examination (MMSE) ≥ 18.

The exclusion criteria included age < 18 years, missing informed consent form, missing postoperative neuropsychological testing, other histopathological diagnosis (e.g., malignant intracranial process), and preoperative MMSE < 18.

### Study design

Patients were included in this prospective study after detailed information and informed consent were obtained. Neuropsychological testing (including the basic test battery [MMSE] and the extended test battery [as explained below] was performed at 3 time points by medical students supervised by a certified neuropsychologist: preoperatively (t_0_), immediately postoperatively during inpatient stay (t_1_), and during follow-up at the first outpatient checkup (t_2_).

### Basic test battery

The basic test battery was the MMSE^29^. This well-known test was used to examine the patients’ basic cognitive functions.

### Extended test battery

The extended test battery comprised 3 categories of cognitive functions: attention, memory, and executive functions. The tests used to evaluate these 3 categories are described below.

#### Attention

The computer-based Test of Attentional Performance (TAP) was used to evaluate cognitive functions in the *attention* category (in addition to the TMT-A)^30^.

### Alertness

Reaction times (shown in ms) for visual stimuli were recorded in this subtest, either with acoustic notification (alertness W_sound) or without acoustic notification (W_O_sound).

### Divided attention

Simultaneous reaction times (ms) to visual (divided attention visual) and to auditory (divided attention auditory) stimuli were measured. Furthermore, the numbers of mistakes (divided attention failure) and of omissions (divided attention selected) were recorded.

### Visual field

Visual stimuli were provided while the patients fixated at a central point. The data (reaction time [ms] and omissions [skip]) are shown separately for the right/left and central visual fields.

### Trail-Making-Test A

The Trail-Making-Test A (TMT-A) task was to connect numbers (1–25) in the right order as fast as possible. Time is measured and shown in seconds^31^.

#### Memory

##### Wechsler Memory Scale (WMS)

The Wechsler Memory Scale Revised^32^ is a test battery for assessing different aspects of memory and includes 13 subtests. In this study, the block- and digit-span subtests were used to evaluate verbal and nonverbal short-term memory. A row of digits was read out, or a group of wooden blocks was arranged in a specific order. Patients were asked to repeat the tasks immediately (memory span verbal/nonverbal [ms v/ms nv]) and after a delay (working memory verbal/nonverbal [wm v/wm nv]).

### Verbal learning and memory test

The Verbal Learning and Memory Test (VLMT) was used to analyze episodic memory. A learning list of 15 words was read out 5 times, and patients were asked to repeat the words after the words were read out once (Dg1) and after being read out 5 times (Dg5). The gained knowledge was measured (Dg1-5). An interference list with different words was read out, and the patients again were asked to repeat the words from the learning list immediately (Dg6) and after a delay of 30 min (Dg7). The loss of knowledge was measured after the interference list was read (Dg5-6) and after a delay (Dg5-7).

### Rey Osterrieth complex figure test (ROCF)

The well-known neuropsychological Rey Osterrieth Complex Figure Test (ROCF) was used to analyze the patients’ visual memory and visual constructive capacity^33^ and included 3 subtests. Initially, the subjects were asked to copy a geometrical figure that was shown to them. This first subtest was not assessed in the present study. In the next 2, subtests, which were analyzed in this study, the subjects were asked to draw a geometrical figure shown to them before, both immediately (ROCF copy) and after a 30-min delay (ROCF delay).

#### Executive functions

##### Trail-Making-Test B

The Trail-Making-Test B (TMT-B) was similar to the TMT-A; however, the patients were asked to connect letters and numbers in alternation (eg, 1-A-2-B)^31^. The time needed for correct connection was measured in seconds.

### Regensburg word fluency test

Formal lexical and semantic fluency was measured by the Regensburg Word Fluency Test (RWT)^34^. Words with a specific initial letter (eg, words beginning with the letter A or L) were requested for formal lexical fluency, and words in a specific category (eg, in the category *animals*) were requested for semantic fluency. Another subtest was used to analyze the capability to change between both categories.

### Stroop word color test

Color-word interference was analyzed with the Stroop Word Color Test^35^. The first subtest was word reading, in which the patients were asked to read words (green, blue, etc.) written in black. The second subtest was line naming, in which the colors of different lines were recorded. In the third and last subtest (*interference*), patients were asked to read out the color of words that were written in different colors (eg, the word “green” was written in blue).

### Assessment of mood and pain

Patients’ mood was assessed by the well-known Beck Depression Inventory-II (BDI-II) in 52/58 patients at t_0_, in 37/58 patients at t_1_, and in 19/24 at t_2_^36^. The BDI-II is scored from 0 to 63, with higher scores for higher extent of depression. The median score of the normative population was 7.4 (population of n = 582 depressive patients and n = 260 healthy controls, according to the manual of Hautziger et al.^37^ The participants were divided into 5 groups according to scores from the BDI-II: no depression (0–8), minimal depression (9–13), slight depression (14–9), moderate depression (20–28), and severe depression (> 28).

Pain, especially headache, in this cohort was assessed using the IBK, the German version of the Headache Disability Inventory (HDI)^38^. This test was available for 43/58 patients at t_0_, 33/58 patients at t_1_, and 18/24 patients at t_2_. Pain was divided into 4 scales: no headache, slight headache, moderate headache, and severe headache.

### Volumetric measurement

A neuroradiologist performed manual segmentation of the contrast-enhancing part of the intracranial lesion pre- and postoperatively (iPlan Net Cranial 3.0, Brainlab AG, Munich, Germany). No volumetric measurement was performed of vascular lesions or trigeminal neuralgia.

### Surgery

Surgery was performed at the Department of Neurosurgery with the aim of maximum tumor resection in patients with benign tumors. Pituitary adenomas were resected using a transnasal-transsphenoidal approach, and other benign tumors were resected using trepanation. Aneurysms were treated by clipping and pterional trepanation, whereas trigeminal nerve neuralgia was treated by microvascular decompression.

### Statistical analysis

IBM SPSS Statistics versions 24.0, 25.0, and 26.0 (SPSS Inc., IBM Corp., Armonk, NY, USA) was used for the statistical analysis. Normally distributed data were shown as means/standard deviations, and non-normally distributed data were shown as medians/interquartile range (IQR). The delta between the pre- and postoperative percentile ranks was recorded, and the patients were divided into improvement/stable condition or deterioration groups. The Wilcoxon test for paired samples was used for pre- and postoperative comparisons of mood and pain and of the basis test battery (MMSE). Binary logistic regression analyses were performed to identify risk factors for postoperative changes of cognitive functions. *P* < 0.05 was defined as significant.

### Ethical approval and informed consent

The study was conducted in accordance with the ethical standards of the 1964 Declaration of Helsinki and its later amendments and approved by the local ethics committee (Ethics committee technical university munich). Informed consent was signed by all study participants.

## Results

### Patient population

Initially, 81 patients were included in the study. Of them, 23 patients were excluded: 22 patients did not perform neuropsychological testing after surgery due to reduced general condition or lack of retrieved informed consent (for further neuropsychological testing after surgery), and 1 patient was excluded due to missing surgery (neuroradiological intervention).

Therefore, 58 patients (21 men, 37 women, mean age 51.6 years [range 24–76]) were included in this study, with meningioma (n = 23, 19/23 WHO grade I, 3/23 WHO grade II, 1/23 WHO grade III), pituitary adenoma (n = 8), vestibular schwannoma (n = 7), neuralgia of the trigeminal nerve (n = 3), cavernoma (n = 3), intracranial aneurysm (n = 4), pineocytoma (n = 2), arterial-venous malformation (n = 2), hemangiopericytoma (n = 1, WHO grade II), clivus chordoma (n = 1), colloidal cyst (n = 1), subependymoma (n = 1), and others (n = 2). Of the 58 lesions, 20 were located in the right hemisphere, 23 were located in the left hemisphere, and 15 were located in the midline (pituitary adenomas, pineocytoma, aneurysm of the basilar artery, chordoma, subependymoma, and 2 meningiomas).

The main tumor locations were the frontal lobe (23/58) and infratentorial region (16/58). The baseline patient characteristics also included information about initial symptoms and adjuvant treatment, as shown in Table 1. During follow-up (at t_2_), 24/58 patients (10 men, 14 women, mean age 46.8 years [range 24–73], with meningioma [n = 8], vestibular schwannoma [n = 3], pituitary adenoma [n = 5], pineocytoma [n = 2], clivus chordoma [n = 1], cavernoma [n = 2], aneurysm [n = 1], neuralgia of the trigeminal nerve [n = 1], and others [n = 1]) underwent neuropsychological testing. The others either were lost to follow-up or withdrew informed consent. The mean time from preoperative testing (t_0_) to surgery was 3.7 days (range 1–23 days), the mean time from surgery to postoperative testing during inpatient stay (t_1_) was 7.6 days (range 2–55 days), and the mean time from surgery to follow-up at the first outpatient control (t_2_) testing was 99.5 days (range 61–197 days).

**Table 1:** Patient characteristics classified according to tumor/lesion.

	Meningioma (n=23)	Pituitary adenoma (n=8)	Vestibular schwannoma (n=7)	Trigeminal neuralgia (n=3)	Vascular (n=9)	Other tumors (n=8)
Age	59.2 years (30–76)	51.1 years (24–68)	55.6 years (38–69)	37.0 years (31–44)	41.8 years (28–61)	43.5 years (30–61)
Sex (female)	18/23	3/8	2/7	3/3	4/9	7/8
**Initial symptom**						
Seizure	2/23	0/8	0/7	0/3	1/9	0/8
Neurological deficit	5/23	2/8	5/7	0/3	2/9	3/8
Pain	3/23	2/8	0/7	3/3	0/9	3/8
Fatigue	2/23	2/8	0/7	0/3	0/9	1/8
Incidental	11/23	2/8	2/7	0/3	6/9	1/8
**KPS**						
t_0_	100 (90–100)	100 (100–100)	100 (100–100)	100 (100–100)	100 (100–100)	100 (85–100)
t_1_	100 (100–100)	100 (100–100)	100 (100–100)	100 (100–100)	100 (70–100)	100 (85–100)
t_2_	100 (90–100)	100 (100–100)	100 (100–100)	n/a	100 (100–100)	100 (85–100)
**Main tumor location**						
Frontal lobe	18/23	0/8	0/7	0/3	5/9	0/8
Temporal lobe	3/23	0/8	0/7	0/3	0/9	2/8
Parietal lobe	1/23	0/8	0/7	0/3	1/9	0/8
Mid line	0/23	8/8	0/7	0/3	0/9	3/8
Infratentorial	1/23	0/8	7/7	3/3	3/9	2/8
Ventricle	0/23	0/8	0/7	0/3	0/9	1/8
**Hemisphere**						
Right	12/23	0/8	4/7	0/3	4/9	0/8
Left	10/23	0/8	3/7	3/3	4/9	3/8
Median	1/23	8/8	0/7	0/3	1/9	5/8
**Education**						
Unknown	2/23	0/8	0/7	0/3	0/9	0/8
None	0/23	0/8	0/7	0/3	0/9	0/8
Main school	8/23	4/8	5/7	2/3	3/9	3/8
Secondary school	7/23	1/8	1/7	1/3	4/9	3/8
A-level	6/23	3/8	1/7	0/3	2/9	2/8
**Tumor volume**						
Preoperative volume	7.0 cm^3^ (2.5–17.5)	2.2 cm^3^ (0.9–6.7)	3.0 cm^3^ (0.4–25.7)	n/a	n/a	0.8 cm^3^ (0.5–1.4)
Postoperative volume	0.0 cm^3^ (0.0–0.5)	0.2 cm^3^ (0.0–0.2)	0.0 cm^3^ (0.1–0.4)	n/a	n/a	0.0 cm^3^ (0.0–0.0)
**Recurrent disease, adjuvant therapy**						
Recurrent disease	5/23	4/8	0/7	0/3	0/9	1/8
Postoperative radiation therapy	3/23	0/8	0/7	0/3	0/9	1/8

### MMSE

No significant differences were observed between preoperative MMSE (29.0 [IQR 28.0–30.0] and postoperative MMSE (29.0 [27.0–30.0]; *P* = 0.278) as well as between preoperative MMSE and follow-up MMSE (28.0 [27.3–29.8]); *P* = 0.522).

### Pre- and postoperative comparisons

Analyses were performed for all patients and the patients in the meningioma and pituitary adenoma subgroups (Table 2). Improvement of cognition was defined as stable/improving cognitive functions in more than 50% of the patients. In the *attention* category, 12/14 subtests showed early postoperative improvement/stable condition at t_1_, as compared to 11/13 subtests in the *memory* category and 6/9 subtests in the *executive functions* category. Similar results were observed in the subgroup of meningioma patients (Table 2). The percentage of patients with improvement during follow-up (t_2_) was similar, comprising 11/14 subtests in the *attention* category and, even higher, 13/13 subtests in the *memory* category and 9/9 subtests in the *executive functions* category. Among the meningioma patients, this rate at t_2_ was lower in the *memory* category (8/13 subtests). In the subgroup of patients with pituitary adenomas, 12/14 subtests improvement/stable condition immediately postoperatively (t_1_) in the *attention* category, as compared to 7/13 subtests in the *memory* category and 6/9 subtests in the *executive functions* category. At t_2_, 12/14 subtests in the *attention* category showed improvement/stable condition, compared to 12/13 subtests in the *memory* category and 7/9 subtests in the *executive functions* category (Table 2).

**Table 2:** Patients with improvement of cognition and/or stable cognition after surgery.

Test	All patients		Meningioma		Pituitary adenoma	
	Improvement stable condition at t_1_	Improvement stable condition at t_2_	Improvement stable condition at t_1_	Improvement stable condition at t_2_	Improvement stable condition at t_1_	Improvement stable condition at t_2_
**Attention**						
Alertness W_O_sound	24/53 (45.3%)	12/24 (50.0%)	7/21 (33.3%)	4/8 (50.0%)	4/8 (50%)	1/5 (20.0%)
Alertness W_sound	23/53 (43.4%)	*16/23 (69.6%)*	*11/21 (52.4%)*	*6/8 (75.0%)*	4/8 (50.0%)	*3/5 (60.0%)*
Alertness phasic	*29/53 (54.7%)*	*16/23 (69.6%)*	*13/21 (61.9%)*	*7/8 (87.5%)*	*5/8 (62.5%)*	*3/5 (60.0%)*
Divided attention visual	*33/53 (62.3%)*	*18/22 (81.8%)*	*12/21 (57.1%)*	*7/8 (87.5%)*	*5/8 (62.5%)*	*3/5 (60.0%)*
Divided attention auditive	*27/49 ((55.1%)*	*12/20 (60.0%)*	*11/18 (61.1%)*	*4/6 (66.7%)*	*5/8 (62.5%)*	*4/5 (80.0%)*
Divided attention failure	*33/53 (62.3%)*	11/22 (50.0%)	*13/21 (61.9%)*	4/8 (50.0%)	*7/8 (87.5%)*	*3/5 (60.0%)*
Divided attention selected	*32/53 (60.4%)*	*13/22 (59.1%)*	*13/21 (61.9%)*	*6/8 (75.0%)*	*5/8 (62.5%)*	*3/5 (60.0%)*
Visual field right	*29/51 (56.9%)*	*13/21 (61.9%)*	8/20 (40.0%)	*4/7 (57.1%)*	*6/8 (75.0%)*	*3/5 (60.0%)*
Visual field skip_right	*32/51 (62.7%)*	*11/21 (52.4%)*	*13/20 (65.0%)*	*4/7 (57.1%)*	*7/8 (87.5%)*	*3/5 (60.0%)*
Visual field left	*28/50 (56.0%)*	*13/21 (61.9%)*	*11/19 (57.9%)*	*4/7 (57.1%)*	*6/8 (75.0%)*	*4/5 (80.0%)*
Visual field skip_left	*30/51 ((58.8%)*	*12/21 (57.1%)*	*12/20 (60.0%)*	*4/7 (57.1%)*	*6/8 (75.0%)*	*3/5 (60.0%)*
Visual field central	*27/50 (54.0%)*	10/21 (47.6%)	*12/19 (63.2%)*	*4/7 (57.1%)*	*6/8 (75.0%)*	*3/5 (60.0%)*
Visual field skip_central	*28/51 (54.9%)*	*12/21 (57.1%)*	10/20 (50.0%)	*5/7 (71.4%)*	*5/8 (62.5%)*	2/5 (40.0%)
TMT-A	*42/56 (75.0%)*	*16/20 (80.0%)*	*14/23 (60.9%)*	*6/7 (85.7%)*	*8/8 (100.0%)*	*5/5 (100.0%)*
**Memory**						
WMS ms v	*38/57 (66.7%)*	*19/23 (82.6%)*	*14/23 (60.9%)*	*7/8 (87.5%)*	*6/8 (75.0%)*	*3/5 (60.0%)*
WMS wm v	*35/57 (61.4%)*	*18/23 (78.3%)*	*13/23 (56.5%)*	*6/8 (75.0%)*	*6/8 (75.0%)*	*3/5 (60.0%)*
WMS ms nv	*39/57 (68.4%)*	*18/23 (78.3%)*	*19/23 (82.6%)*	*6/8 (75.0%)*	4/8 (50.0%)	*4/5 (80.0%)*
WMS wm nv	*31/57 (54.4%)*	*18/23 (78.3%)*	*13/23 (56.5%)*	*8/8 (100.0%)*	4/8 (50.0%)	*3/5 (60.0%)*
VLMT Dg1	*34/58 (58.6%)*	*14/23 (60.9%)*	*14/23 (60.9%)*	*5/8 (62.5%)*	*6/8 (75.0%)*	2/5 (40.0%)
VLMT Dg5	*35/58 (60.3%)*	*15/23 (65.2%)*	*12/23 (52.2%)*	4/8 (50.0%)	*7/8 (87.5%)*	*3/5 (60.0%)*
VLMT Dg1_5	*35/58 (60.3%)*	*15/23 (65.2%)*	*12/23 (52.2%)*	4/8 (50.0%)	*7/8 (87.5%)*	*3/5 (60.0%)*
VLMT Dg6	*32/58 (55.2%)*	*16/23 (69.6%)*	*12/23 (52.2%)*	3/8 (37.5%)	4/8 (50.0%)	*5/5 (100.0%)*
VLMT Dg7	28/58 (48.3%)	*15/23 (65.2%)*	11/23 (47.8%)	4/8 (50.0%)	4/8 (50.0%)	*4/5 (80.0%)*
VLMT Dg5_6	*34/58 (58.6%)*	*15/23 (65.2%)*	*12/23 (52.2%)*	4/8 (50.0%)	4/8 (50.0%)	*4/5 (80.0%)*
VLMT Dg5_7	24/58 (41.4%)	*13/23 (56.5%)*	11/23 (47.8%)	*5/8 (62.5%)*	4/8 (50.0%)	*4/5 (80.0%)*
ROCF copy	*42/56 (75.0%)*	*21/23 (91.3%)*	*18/23 (78.3%)*	*6/8 (75.0%)*	*7/8 (87.5%)*	*5/5 (100.0%)*
ROCF delay	*53/56 (94.6%)*	*22/22 (100.0%)*	*21/23 (91.3%)*	*8/8 (100.0%)*	*8/8 (100.0%)*	*5/5 (100.0%)*
**Executive functions**						
TMT-B	*42/55 (76.4%)*	*18/20 (90.0%)*	*19/22 (86.4%)*	*6/7 (85.7%)*	*5/8 (62.5%)*	*5/5 (100.0%)*
Stroop’s word reading	*29/57 (50.9%)*	*12/22 (54.5%)*	9/23 (39.1%)	*5/8 (62.5%)*	*7/8 (87.5%)*	2/5 (40.0%)
Stroop’s naming	28/57 (49.1%)	*13/22 (59.1%)*	8/23 (34.8%)	*5/8 (62.5%)*	*5/8 (62.5%)*	*3/5 (60.0%)*
Stroop’s interference	*31/57 (54.4%)*	*16/22 (72.7%)*	10/23 (43.5%)	*7/8 (87.5%)*	*5/8 (62.5%)*	*3/5 (60.0%)*
Stroop’s failure	*45/57 (78.9%)*	*17/22 (77.3%)*	*18/23 (78.3%)*	*6/8 (75.0%)*	*7/8 (87.5%)*	*4/5 (80.0%)*
RWT lexical	*35/58 (60.3%)*	*18/23 (78.3%)*	*12/23 (52.2%)*	*6/8 (75.0%)*	*6/8 (75.0%)*	*3/5 (60.0%)*
RWT semantic	28/58 (48.3%)	*14/23 (60.9%)*	9/23 (39.1%)	*6/8 (75.0%)*	3/8 (37.5%)	*5/5 (100.0%)*
RWT turning lexical	*36/58 (62.1%)*	*16/23 (69.6%)*	*15/23 (65.2%)*	*8/8 (100.0%)*	4/8 (50.0%)	2/5 (40.0%)
RWT turning semantic	21/58 (36.2%)	*17/23 (73.9%)*	8/23 (34.8%)	*7/8 (87.5%)*	2/8 (25.0%)	*4/5 (80.0%)*

Data are shown as numbers of patients with improvement/stable condition of cognitive functions in relation to all patients performing this subtest immediately after surgery and during follow-up. Improvement > 50% shown in italics.

**Figure 1:**
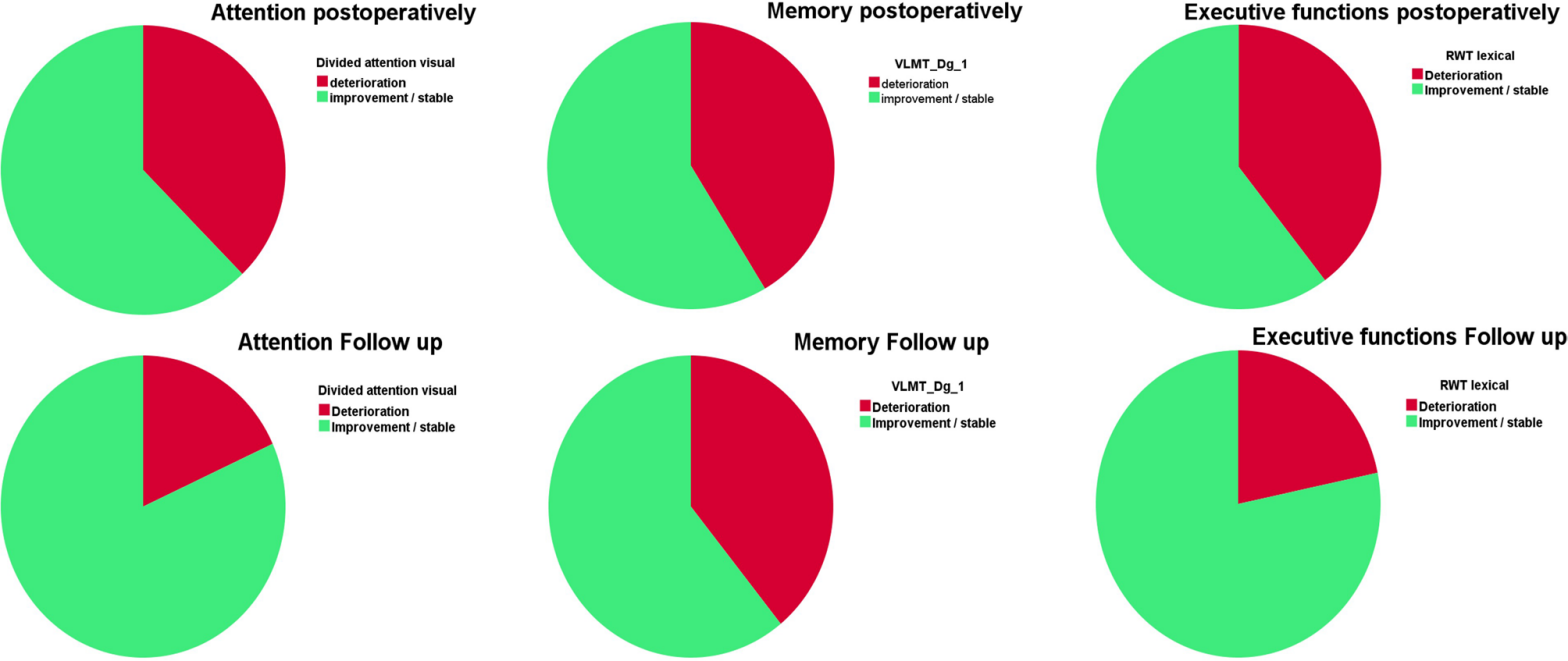
Proportion of patients with improvement/stable condition and deterioration at t_1_ and t_2_ in selected subtests of the *attention* (left), *memory* (middle), and *executive functions* (right) categories.

Figure 1 shows the distributions of improvement and deterioration in the subtests of the *attention, memory*, and *executive functions* categories at t_1_ and t_2_.

**Figure 2:**
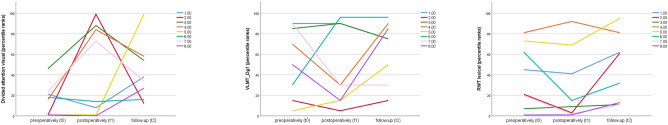
Plots for the 8 meningioma patients in selected subtests of the *attention* (left), *memory* (middle), and *executive functions* (right) categories.

Figure 2 presents the results of the 8 meningioma patients at different time points in each category (*attention*, *memory*, and *executive functions*).

### Mood and pain

**Figure 3:**
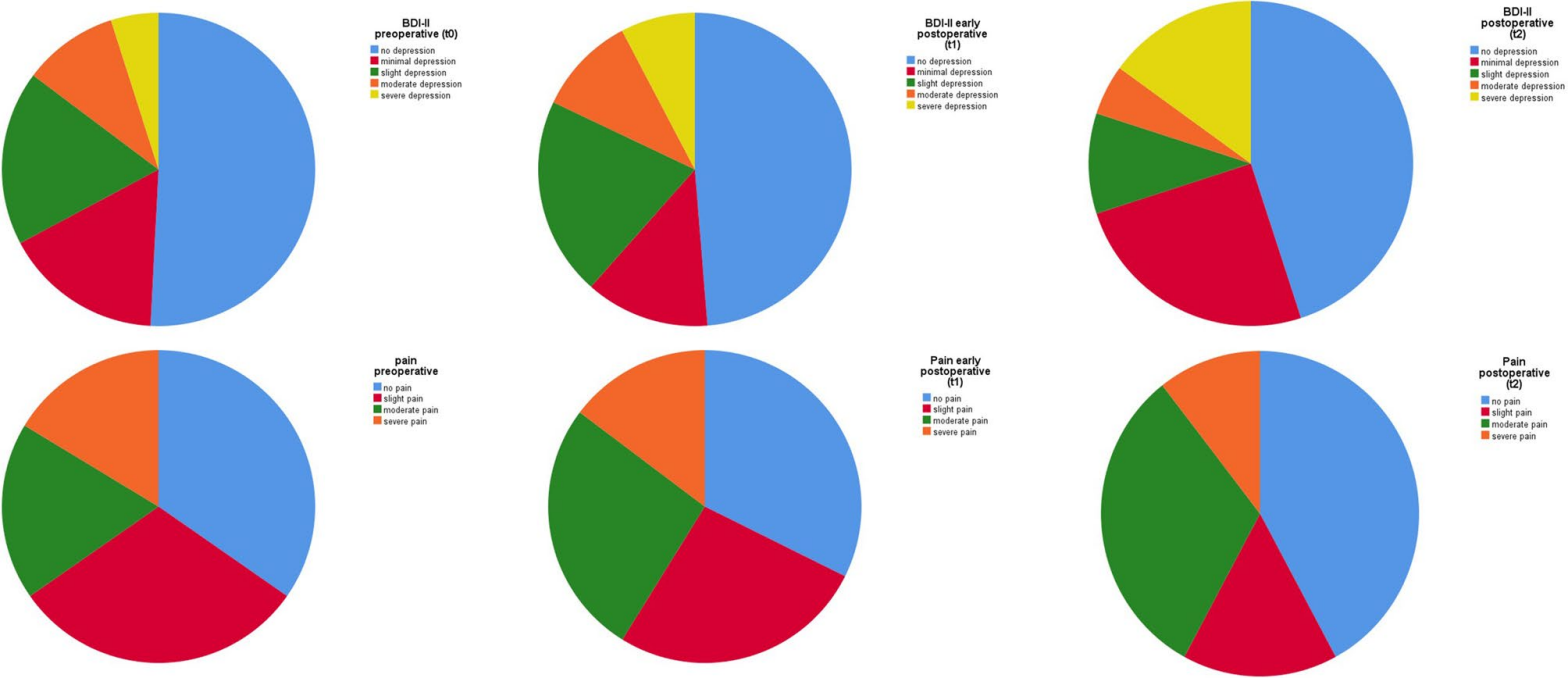
Distributions of mood (BDI-II) and pain before and after surgery.

Mood did not change significantly after surgery at t_1_ (*P* = 0.484) and at t_2_ (*P* = 0.306). In addition, pain did not change significantly at t_1_ (*P* = 0.060) or at t_2_ (*P* = 0.564). The distributions of depression and pain at the different time points are shown in Fig. 3.

### Multivariate analysis

For the homogenous subgroup of meningioma patients, binary logistic regression analyses were performed with change of cognition after surgery as the dependent variable and age, KPS, and tumor volume as the independent variables. Odds ratios (95% CI) are presented in Table 3. No significant influencing factors could be identified in this analysis.

**Table 3:** Binary logistic regression analysis for meningioma patients.

	Covariates		
	Age	KPS	Lesion volume
**Attention**			
Alertness W_O_sound	1.062 (0.980–1.152)	1.134 (0.926–1.390)	1.039 (0.973–1.109)
Alertness W_sound	1.019 (0.955–10.86)	1.008 (0.907–1.119)	1.003 (0.956–1.053)
Alertness phasic (p)	0.995 (0.932–1.061)	0.865 (0.673–1.113)	0.957 (0.889–1.030)
Divided attention visual (ms)	1.024 (0.959–1.093)	1.023 (0.920–1.138)	0.991 (0.941–1.042)
Divided attention auditive (ms)	1.037 (0.969–1.110)	0.942 (0.768–1.156)	0.981 (0.919–1.048)
Divided attention failure (n)	1.016 (0.952–1.084)	0.975 (0.856–1.111)	1.016 (0.956–1.080)
Divided attention selected (n)	1.013 (0.949–1.081)	0.976 (0.866–1.100)	0.978 (0.929–1.031)
Visual field right (ms)	1.008 (0.941–1.081)	1.024 (0.917–1.143)	1.053 (0.982–1.130)
Visual field skip_right (n)	0.937 (0.848–1.036)	1.035 (0.912–1.176)	0.973 (0.919–1.030)
Visual field left (ms)	1.018 (0.951–1.091)	1.074 (0.939–1.227)	0.987 (0.922–1.056)
Visual field skip_left (n)	0.953 (0.885–1.027)	0.965 (0.862–1.082)	1.015 (0.954–1.081)
Visual field central (ms)	0.977 (0.909–1.050)	1.021 (0.918–1.136)	1.021 (0.953–1.095)
Visual field skip_central (n)	1.000 (0.939–1.066)	1.003 (0.903–1.114)	1.011 (0.962–1.062)
TMT-A (s)	1.015 (0.954–1.080)	0.992 (0.890–1.105)	0.976 (0.926–1.030)
**Memory**			
WMS ms v (p)	0.993 (0.933–1.056)	0.994 (0.894–1.105)	1.002 (0.953–1.053)
WMS wm v (p)	1.007 (0.948–1.070)	0.957 (0.846–1.082)	0.974 (0.924–1.027)
WMS ms nv (p)	0.979 (0.898–1.066)	0.980 (0.836–1.150)	1.027 (0.929–1.136)
WMS wm nv (p)	0.983 (0.922–1.049)	1.038 (0.931–1.156)	1.043 (0.974–1.118)
VLMT Dg1 (p)	1.036 (0.963–1.115)	1.469 (0.916–2.357)	1.102 (0.967–1.256)
VLMT Dg5 (p)	0.927 (0.840–1.023)	5.761 (0.000–n.a.)	1.060 (0.922–1.219)
VLMT Dg1_5 (p)	0.933 (0.862–1.010)	1.026 (0.907–1.160)	1.014 (0.958–1.073)
VLMT Dg6 (p)	0.951 (0.886–1.021)	1.043 (0.918–1.185)	1.023 (0.966–1.083)
VLMT Dg7 (p)	0.958 (0.897–1.024)	1.031 (0.913–1.165)	1.019 (0.965–1.076)
VLMT Dg5_6 (p)	0.963 (0.892–1.040)	1.219 (0.821–1.810)	1.147 (0.999–1.317)
VLMT Dg5_7 (p)	0.976 (0.915–1.041)	1.022 (0.918–1.139)	1.050 (0.977–1.129)
ROCF copy (p)	1.015 (0.940–1.095)	1.054 (0.940–1.183)	0.994 (0.944–1.047)
ROCF delay (p)	3.224 (0.000–n.a.)	8.940 (0.000–n.a.)	0.856 (0.000–n.a.)
**Executive functions**			
TMT-B (s)	1.050 (0.959–1.149)	0.945 (0.691–1.293)	0.950 (0.863–1.047)
Stroop’s word reading (s)	1.063 (0.985–1.147)	1.066 (0.942–1.207)	1.016 (0.962–1.072)
Stroop’s naming (s)	1.013 (0.951–1.080)	1.041 (0.918–1.179)	1.015 (0.964–1.068)
Stroop’s interference (s)	1.067 (0.992–1.148)	1.170 (0.939–1.459)	1.041 (0.972–1.115)
Stroop’s failure (p)	1.100 (0.992–1.220)	1.196 (0.984–1.453)	1.010 (0.947–1.076)
RWT lexical (p)	1.052 (0.984–1.125)	1.171 (0.948–1.446)	1.050 (0.983–1.122)
RWT semantic (p)	0.982 (0.923–1.045)	0.958 (0.861–1.065)	0.978 (0.927–1.032)
RWT turning lexical (p)	1.003 (0.941–1.069)	0.997 (0.881–1.129)	1.048 (0.958–1.147)
RWT turning semantic (p)	1.025 (0.960–1.095)	5.944 (0.000–n.a.)	1.042 (0.930-1.167)

Data of independent variables presented as odds ratio (95% CI).

*KPS* Karnofsky Performance Status Scale (postoperative values); Tumor volume (cm^3^) as preoperative volume.

## Discussion

Cognitive functions in patients with benign intracranial lesions improved or remained stable immediately postoperatively and during follow-up among the majority of patients in all categories of cognitive functions for the cohort of 58 patients. No significant influencing factors, like age, KPS, or tumor volume, were identified for changes in cognitive functions among meningioma patients.

The preoperative neurocognitive functions of patients with benign intracranial lesions were analyzed in a previous study, which showed that age and KPS were the main risk factors for impaired neurocognitive functions before operation^28^. In this study, we analyzed a subgroup of patients with available, extensive postoperative neurocognitive testing.

This study showed improvement or stable condition of cognitive functions in the *attention*, *memory*, and *executive functions* categories. These results agree with those of Tucha et al., who studied (elderly) meningioma patients, showing postoperative improvement mainly in the *attention* and *memory* domains and no deterioration of preoperative cognitive functions^15,17,39^. Another recent study be Meskal et al. showed postoperative improvement in almost all cognitive domains, except for psychomotor speed and reaction time^16^.

The mentioned studies by Tucha et al. reported no improvement in *executive functions* after meningioma surgery^17,39^. A recent previous study on meningioma patients also reported postoperative improvement of cognitive functions but with lower ongoing cognitive scores as compared to healthy controls^40^.

The rate of improvement/stable condition was even higher during follow-up (t_2_), as compared to immediate postoperative testing (t_1_), in this cohort. These results might be explainable by postoperative edema or reduced postoperative functional independence (KPS), a known risk factor for cognitive impairment^7^. Previous studies also showed a transient decline of cognition with recovery at follow-up after surgery and after irradiation^15,19,41^. The time point when postoperative evaluation occurs might be important for these findings. In this study, the first postoperative testing was performed quite early (mean time from surgery to t_1_ of 7.6 days). Thus, the aftereffects of surgery might be more prominent at this date. In the other mentioned studies on meningioma patients, postoperative testing was performed later (about 3 months after surgery), which agrees with our follow-up examination (t_2_)^15,17,39^.

However, the results of the follow-up analyses should be taken with caution. According to the study design (t_1_ = postoperative testing during inpatient stay; t_2_ = first follow-up testing on outpatient controls), there were major differences in the time periods, with some overlapping results. Furthermore, only patients with a MMSE > 18 underwent extensive neuropsychological testing, thus only selecting patients without major deterioration after surgery.

The highest rate of improvement/stable condition was observed in the ROCF delay subtest, at almost 100%. This improvement might have been because the patients remembered that they had to perform the same task in 30 min. Therefore, these results should be considered with caution.

In contrast to other studies focusing mainly on 1 entity (eg, meningioma or pituitary adenoma), this study included different entities and also vascular lesions. This might introduce a bias due to the high majority of the diseases. However, considering a more heterogeneous group could also provide additional information, as compared to focusing on only 1 entity.

Among the meningioma patient subgroup, tumor size was not observed as a significant influencing factor, contrasting a previous study by Liouta et al.^14^ These differences might be explained by the different study designs. The present study focused on patients with benign intracranial lesions and with therefore had a lower number of meningioma patients, whereas Liouta et al. focused only on meningioma patients and therefore had a larger number of patients.

The surgical approach and tumor location might affect cognitive functions. Due to the low number of patients in the present study cohort, no further analyses were conducted regarding these possibilities. Surgery was performed according to the neurosurgical standards at our department and did not significantly differ between the patient subgroups.

This study has several limitations. The high dropout rate during follow-up (at t_2_) was a main limitation and might have introduced unavoidable bias. Patients with less cognitive deficits might be more likely to perform cognitive testing during follow-up than patients with considerable restrictions would. This might have biased the results and resulted in overestimation of the rate of patients without deterioration after surgery. Further studies with a lower dropout rate are necessary to address this.

Additional limitations include the low numbers of patients with some diseases and the variety of diseases included in this cohort. However, subependymoma, clivus chordoma, arterial-venous malformation, and cavernoma are rare intracranial lesions, and to our knowledge, cognitive functions have not been assessed before in patients with these lesions. To address this, a subgroup analysis was performed with meningioma patients only.

In particular, the number of unruptured aneurysms was very low in this cohort (n = 4), and the study does not add any new findings to the already known results of the ISAT trial, which showed cognitive improvement after endovascular treatment of aneurysms^24^.

The study population remains a very heterogeneous population with small numbers of each individual pathology. The significance of this study might be limited due to this heterogeneous cohort and many other possible confounding variables that would affect cognitive outcomes. However, the aim of this prospective study was to include all types of benign intracranial lesions and not to select special subgroups (eg, meningioma patients) as previous studies on such cohorts already exist. This exploratory study might draw attention to this heterogeneous patient cohort and might be of interest for further (prospective) studies.

## Conclusions

Cognitive functions improved or remained stable in the *attention*, *memory*, and *executive functions* categories after surgery of benign intracranial lesions in the majority of our cohort of 58 patients. Due to the high dropout rate and the various intracranial lesions included in this study, the results of this study should be taken with caution, and further studies are necessary to confirm the results.
